# Tanshinone IIA pretreatment protects free flaps against hypoxic injury by upregulating stem cell-related biomarkers in epithelial skin cells

**DOI:** 10.1186/1472-6882-14-331

**Published:** 2014-09-04

**Authors:** Zihan Xu, Lijun Wu, Yaowen Sun, Yadong Guo, Gaoping Qin, Shengzhi Mu, Ronghui Fan, Benfeng Wang, Wenjie Gao, Zhenxin Zhang

**Affiliations:** Department of Burns and Plastic Surgery, Shaanxi Provincial People’s Hospital, 256 West Youyi Road, Xi’an, 710068 China; Department of Plastic Surgery, The Second Affiliated Hospital of Soochow University, Suzhou, 215004 China

**Keywords:** Free flap, Epithelial cell, Traditional Chinese medicine, Wnt, Stem cell

## Abstract

**Background:**

Partial or total flap necrosis after flap transplantation is sometimes encountered in reconstructive surgery, often as a result of a period of hypoxia that exceeds the tolerance of the flap tissue. The purpose of this study was to determine whether Tanshinone IIA (TSA) pretreatment can protect flap tissue against hypoxic injury and improve its viability.

**Methods:**

Primary epithelial cells isolated from the dorsal skin of mice were pretreated with TSA for 2 weeks. Cell Counting Kit-8 and Trypan Blue assays were carried out to examine the proliferation of TSA-pretreated cells after exposure to cobalt chloride. Polymerase chain reaction and western blot analysis were used to assess the expression of β-catenin, vascular endothelial growth factor (VEGF), sex determining region Y-box 2 (SOX2), OCT4 (also known as POU domain class 5 transcription factor 1), Nanog, and glycogen synthase kinase-3 beta (GSK-3β) in TSA-treated cells. In other experiments, after mice were pretreated with TSA for 2 weeks, a reproducible ischemic flap model was implemented, and the area of surviving tissue in the transplanted flaps was measured. Immunohistochemistry was conducted to examine Wnt signaling as well as stem cell- and angiogenesis-related biomarkers in epithelial tissue *in vivo*.

**Results:**

Epidermal cells, pretreated with TSA, showed enhanced resistance to hypoxia. Activation of the Wnt signaling pathway in TSA-pretreated cells was characterized by the upregulation of β-catenin and the downregulation of GSK-3β. The expression of SOX2, Nanog, and OCT4 were also higher in TSA-pretreated epithelial cells than in control cells. In the reproducible ischemic flap model, pretreatment with TSA enhanced resistance to hypoxia and increased the area of surviving tissue in transplanted flaps. The expression of Wnt signaling pathway components, stem-cell related biomarkers, and VEGF and CD34, which are involved in the regeneration of blood vessels, was also upregulated in TSA-pretreated flap tissue.

**Conclusions:**

TSA pretreatment protects free flaps against hypoxic injury and increases the area of surviving tissue by activating Wnt signaling and upregulating stem cell-related biomarkers.

## Background

Flaps are routinely used in plastic surgery for covering tissue defects resulting from trauma, ablative surgery, or congenital malformation, although partial or total flap necrosis after flap surgery is not uncommon [1, 2]. The transplantation of marrow mesenchymal cells [3] and adipose-derived stem cells [4] is effective in regenerating tissue, and Yi et al. [5] report that vascular endothelial growth factor (VEGF) gene therapy can enhance the survival and quality of grafted fat tissue in nude mice. It is also reported that hyperbaric O_2_ is clinically effective as an adjuvant therapy for improving wound healing and neovascularization and attenuating oxidative stress [6]. At the same time, however, there are still clear potential disadvantages of these treatment strategies. Considering hyperbaric O_2_, there is a risk for O_2_ toxicity [7], progressive myopia [8], and grand mal seizures [8] after treatment. Furthermore, more research is needed into stem cell transplantation and gene therapy before experimental results can be translated into broad clinical application [9, 10]. As flap transplantation is becoming more widely used in reconstructive surgery, it is important to understand the mechanism of flap necrosis so that it can be prevented.

Ischemia occurs when there is inadequate blood flow to a specific tissue area after flap reconstruction, and it is an underlying cause of tissue hypoxia and flap loss [11]. When hypoxia exceeds the tolerance level of epithelial cells, necrosis or scarring of flap tissue occurs. Although not all epithelial skin cells exhibit the same resilience to hypoxia, they are a promising source of stem cells that are resistant to noxious surroundings, exhibit anti-apoptotic characteristics, and possess enhanced DNA repair machinery, which are essential properties for cell regeneration and repair [12]. Stem cells are defined by their capacity for self-renewal and their resistance to maladaptive microenvironments, including hypoxia, and there is now compelling evidence that multipotent stem cells contribute to recovery from hypoxia and epidermal repair [13, 14].

Recent advances in our understanding of epithelial stem cells, together with explorations of stem cell-related signaling pathways that control the regeneration of skin, open a new door for improvements in flap surgery. Wnt signaling, which is required for tissue repair and regeneration, is involved in the growth of various cell types and plays a critical role in stem cell maintenance and differentiation [15, 16]. SOX2, Nanog, and OCT4, which are regulated by Wnt signaling, are markers of somatic cell stemness [17]. Takahashi et al. [18] induced pluripotent stem cells by transducing adult human dermal fibroblasts with defined factors such as OCT4 and SOX2. When Wnt signaling is reduced, its associated biomarkers, such as OCT4, SOX2, and β-catenin, are downregulated, and cell resistance to hypoxia and capacity for regeneration are also impaired [19, 20]. For instance, in the zebrafish eye, the inhibition of Wnt signaling results in an abrupt cessation of the normally continuous regeneration of the retina [21]. Also, inhibition of Wnt signaling during skin wounding in mice prevents the formation of epithelial appendages, including hair and sweat glands, which results in prominent scarring of the epidermis [22]. By contrast, elevating Wnt signaling within the wound site promotes the growth of adult skin [22]. Several studies also show that increasing Wnt signaling stimulates healing of many different injuries, including bone fractures, retinal damage, skin wounding, and myocardial infarction [23]. If Wnt signaling is required for tissue regeneration, can we find a convenient and effective way to elevate Wnt signaling in epithelial skin cells with limited regenerative capacity to improve the healing response?

Tanshinone IIA (TSA) is the most abundant diterpene quinone isolated from Danshen (*Salvia miltiorrhiza*) and has been used to treat cardiovascular diseases for more than 2000 years in China. Over the last decade, interest in the mechanism of its versatile protective effects in neurodegenerative diseases, metabolic abnormalities, and ischemic damage has been growing [24–30]. For instance, Zhang et al. demonstrated that TSA enhances cell resistance to hypoxic insult by upregulating miR-133 expression through activation of the mitogen-activated protein kinase (MAPK)/extracellular signal-related kinase (ERK) pathway in neonatal cardiomyocytes [31]. Chen et al. showed that TSA has neuroprotective effects against ischemia/reperfusion (I/R) injury through the inhibition of macrophage migration inhibitory factor (MIF) and the release of tumor necrosis factor-α and interleukin-6 in rats [24]. Furthermore, Zhu et al. found that TSA protects rat primary hepatocytes against carbon tetrachloride toxicity via inhibiting mitochondria permeability transition [32].

In the present study, we investigated whether TSA could protect free flaps against hypoxia-induced necrosis and improve tissue viability in the hypoxic zone. Next, we examined the regulation of the Wnt signaling pathway and stem cell-related biomarkers in epithelial cells and tissue. Finally, we tested whether TSA therapy activates the Wnt signaling pathway and upregulates stem cell-related biomarkers in epithelial cells.

## Methods

### Regents and antibodies

TSA injection (sulfotanshinone sodium injection, 5 mg/mL; First Biochemical Pharmaceutical Co. Ltd., Shanghai, China) was used in the *in vivo* experiments. TSA monomer (Sigma, St. Louis, MO), a lyophilized powder (99.99% purity), which was first dissolved in dimethyl sulfoxide, and then diluted with phosphate-buffered saline (PBS) to the required concentration, was used in the *in vitro* experiments. Antibodies used for immunoblotting and/or immunohistochemistry were as follows: mouse anti-human monoclonal β-catenin (Abcam, Cambridge, MA, USA), rabbit anti-human polyclonal glycogen synthase kinase-3 beta (GSK-3β; Epitomics, Burlingame, CA, USA), rabbit anti-human polyclonal SOX2 (Epitomics, Burlingame, CA, USA), mouse anti-human monoclonal OCT4 (Abcam, Cambridge, MA, USA), mouse anti-human monoclonal Nanog (Abcam, Cambridge, MA, USA), rabbit anti-human polyclonal VEGF (Abcam, Cambridge, MA, USA), mouse anti-human monoclonal CD34 (Abcam, Cambridge, MA, USA), and mouse anti-human monoclonal actin (Beyotime, Shanghai, China).

### Isolation and preparation of epidermal cells

To obtain epithelial cells of high purity, the dorsal skin of male BALB/C mice was processed as previously described with slight modifications [33]. Immediately after mice were killed by cervical dislocation, the shaved dorsal skin was treated for 5 min with a depilatory agent, rinsed under running water, excised, and placed in ice-cold Dulbecco’s Modified Eagle’s Medium (DMEM). The subcutis was removed by scraping with a razor blade. The skin, consisting of dermis and epidermis, was minced with scissors, and the pieces were washed once with DMEM. The pieces of skin were excised and cut into smaller pieces in collagenase buffer containing 0.05% collagenase IV and 0.25% trypsin for 2 h. The digestion solution was filtered through nylon gauze, and the filtrate was sedimented twice by centrifugation at 70 × g for 5 min at 4°C. Finally, cells were collected, counted, and resuspended with DMEM containing 10% fetal bovine serum at a concentration of 5 × 10^5^ in T_25_ flasks. Two days later, cells were digested with 0.25% trypsin and washed gently with PBS to remove fibroblasts resulting from the strong adhesion of epithelial cells. Finally, cells were digested and passaged by repeating the above steps three times to obtain epithelial cells with high purity.

### Cell proliferation assay

Primary epithelial cells were pretreated with TSA (2 mg/L, IC_50_ 98 mg/L, without pro-proliferative effect) for 2 weeks and transferred to glass cover slips and grown to 50% confluence. Cells were exposed to cobalt chloride (CoCl_2_ 50 μmol/L) *in vitro* for 72 h to mimic hypoxia, fixed, permeabilized, and stained with 4’-6-diamidino-2-phenylindole (DAPI) to visualize cell nuclei using fluorescence microscopy (Olympus, Tokyo, Japan).

Other primary epithelial cells were pretreated with TSA (2 mg/L) for 2 weeks, and then exposed to CoCl_2_ (50 μmol/L) *in vitro* for 24, 48, 72, or 96 h. Cell proliferation was assessed using the Cell Counting Kit-8 (CCK-8; Dojindo Molecular technologies, inc, Japan). Results are expressed as the absorbance of each well at 450 nm (OD450). The half maximal inhibitory concentration (IC_50_) of CoCl_2_ was 179 μmol/L for a 72 h treatment time (data not shown). Almost half of the primary epithelial cells died and floated in the culture flasks at this concentration. After several preliminary experiments, a concentration of 50 μmol/L, which is far below the IC_50_, was selected to mimic hypoxia.

### Trypan blue assay

Primary epithelial cells were pretreated with TSA for 2 weeks and exposed to CoCl_2_ (50 μmol/L) for 72 h. Cells were digested and suspended in PBS. Cell suspension solution (0.5 ml) and 0.4% Trypan Blue solution (0.5 ml) were added to a test tube and mixed thoroughly for 5 min to stain non-viable cells blue. Separate counting of viable and non-viable cells was performed, and cell viability (%) was calculated as (viable cells / (viable cells + non-viable cells)) × 100%.

### Quantitative real-time polymerase chain reaction (qRT-PCR)

Total RNA was extracted from TSA-treated primary epithelial cells and their parental cell lines using Trizol Reagent (Invitrogen, Grand Island, NY, USA). Total RNA was reversely transcribed using a Prime Script RT Reagent Kit (TaKaRa, Biotechnology, inc, Dalian, China). mRNA expression was determined by qRT-PCR using SYBR Premix Ex Taq II (TaKaRa). The primers used for the amplification of target genes were as follows:

β-catenin forward primer 5’-ATGGCTACTCAAGCTGAC-3’ and reverse primer 5’-CAGCACTTTCAGCACTCTGC-3’;

GSK-3β forward primer 5’-GTTGGTGGAAATAATAAAGG-3’ and reverse primer 5’-AAGTTGAAGAGGGCAGGT-3’;

SOX2 forward primer 5’-GTGGTGGTACGGGAAATCAC-3’ and reverse primer 5’-TAGCCAGGTTCGAGAATCCA-3’;

OCT4 forward primer 5’-CTGGGTTGATCCTCGGACCT-3’ and reverse primer 5’-CACAGAACTCATACGGCGGG-3’;

Nanog forward primer 5’-AAAGAATCTTCACCTATGCC-3’ and reverse primer 5’-GAAGGAAGAGGAGAGACAGT-3’;

VEGF forward primer 5’-TTACTGCTGTACCTCCACC-3’ and reverse primer 5’-ACAGGACGGCTTGAAGATG-3’; and

β-actin forward primer 5’-CAATGTGGCCGAGGACTTTG-3’ and reverse primer 5’-CATTCTCCTTAGAGAGAAGTGG-3’.

### Western blot analysis

Expression of β-catenin, SOX2, OCT4, Nanog, VEGF, and actin in control and TSA-Treated epithelial cells was detected by western blot analysis as previously described with slight modifications [34]. To prepare whole-cell protein extracts, cells were washed twice with phosphate-buffered saline and then lysed with a modified radio-immunoprecipitation assay buffer (50 mM Tris–HCl pH 7.4, 1% v/v NP-40, 0.25% v/v sodium deoxycholate, 150 mM NaCl, 1 mM EDTA, 1 mM PMSF, 1 mg/mL of protease inhibitors (leupeptin and pepstatin), 1 mM Na_3_VO_4_, and 1 mM NaF) on ice for 30 min. Insoluble material was removed by centrifugation at 12,000 p/min for 15 min at 4°C. The protein concentration of cell lysates was measured using the Bradford Protein Assay Kit (Beyotime, Shanghai, China), and 30 μg of protein samples were loaded on 10% polyacrylamide gels containing sodium dodecyl sulfate and separated by electrophoresis at a constant voltage of 70 V for 2 h and transferred onto 0.45-μm polyvinylidene fluoride membranes (Millipore Corporation, Billerica, MA, USA) at a constant voltage of 100 V for 3 h at 0°C. The membranes were probed with the specific primary antibodies followed by a horseradish peroxidase-conjugate secondary antibody (1:5,000) and detected by enhanced chemiluminescence (ECL kit from Pierce, Rockford, IL, USA). The following primary antibodies were used: β-catenin (dilution 1:1000), SOX2 (dilution 1:400), OCT4 (dilution 1:400), Nanog (dilution 1:500), VEGF (dilution 1:1000), and actin (dilution 1:1000). Unless otherwise indicated, immunoblot reagents were purchased from Beyotime.

### Animals and ethics statement

BALB/c mice (aged 4–6 weeks and weighing approximately 20 g) were obtained from the Chinese Academy of Science and maintained under standard pathogen-free conditions. The experimental protocol was approved by the Shanghai Medical Experimental Animal Care Commission. All surgery was performed under sodium pentobarbital anesthesia, and all efforts were made to minimize suffering.

### *In vivo*evaluation of free flap survival

**Figure 1:**
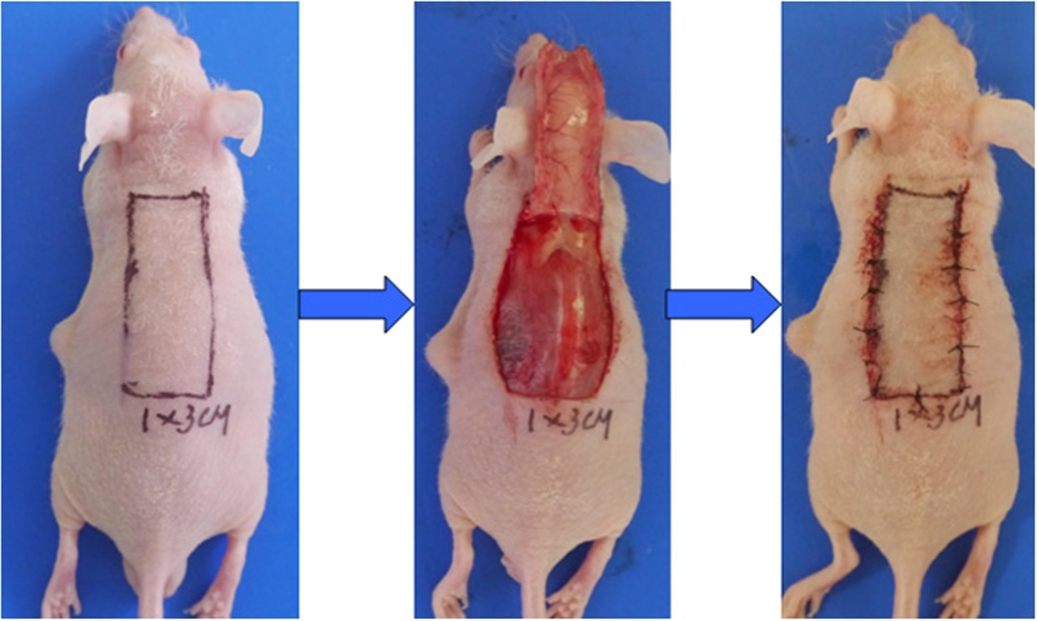
**The reproducible mouse ischemic flap model was implemented using flaps that were 3 cm in length and 1 cm in width, with the main vessels coagulated by cautery.**

BALB/c mice were randomly assigned to the TSA group (n = 6) or the control group (n = 6). TSA mice were injected intraperitoneally with 0.1 ml TSA (10 mg/kg/d) diluted in 5% glucose solution (5% GS) for 14 days. Control mice were injected with 0.1 ml 5% GS. The reproducible ischemic flap model (Figure 1) was implemented as previously described with slight modifications [35]. The dorsal skin area of BALB/c mice was depilated and cleaned, and dorsal random-pattern flaps measuring 1 × 3 cm were constructed. After the flap was elevated, 1 × 0.2 cm epithelial skin tissues in the distal end of the flap were excised for immunohistochemistry, and the main vessels in the flap were coagulated by cautery. Finally, the flap was repositioned and sutured. The percentage of flap survival area was calculated with standard grid paper 7 days after surgery.

### Immunohistochemistry

Immunohistochemical staining of β-catenin, GSK-3 β, SOX2, OCT4, VEGF, and CD34 was carried out using a standard protocol with slight modifications [36]. Epithelial skin tissue was fixed, embedded, and sliced into 5-μm sections. Then, the sections were permeabilized, blocked, and incubated with primary monoclonal antibodies overnight at 4°C. Sections were washed and incubated with anti-mouse or anti-rabbit HRP conjugated secondary antibody (Jackson). Finally, the sections were counterstained with hematoxylin, dehydrated, and cleared with xylene before finally being mounted. The following primary antibodies were used: β-catenin (dilution 1:500), SOX2 (dilution 1:150), OCT4 (dilution 1:200), VEGF (dilution 1:1000), and CD34 (dilution 1:500).

### Statistical analysis

Data are expressed as mean ± standard error of the mean (SEM). Student’s t-tests were used to compare groups. Statistical analysis was performed using SPSS 17.0 software for Windows (SPSS Inc. Chicago, IL, USA). P < 0.05 was considered statistically significant.

## Results

### Epithelial skin cells showed enhanced resistance to hypoxia after TSA pretreatment

**Figure 2:**
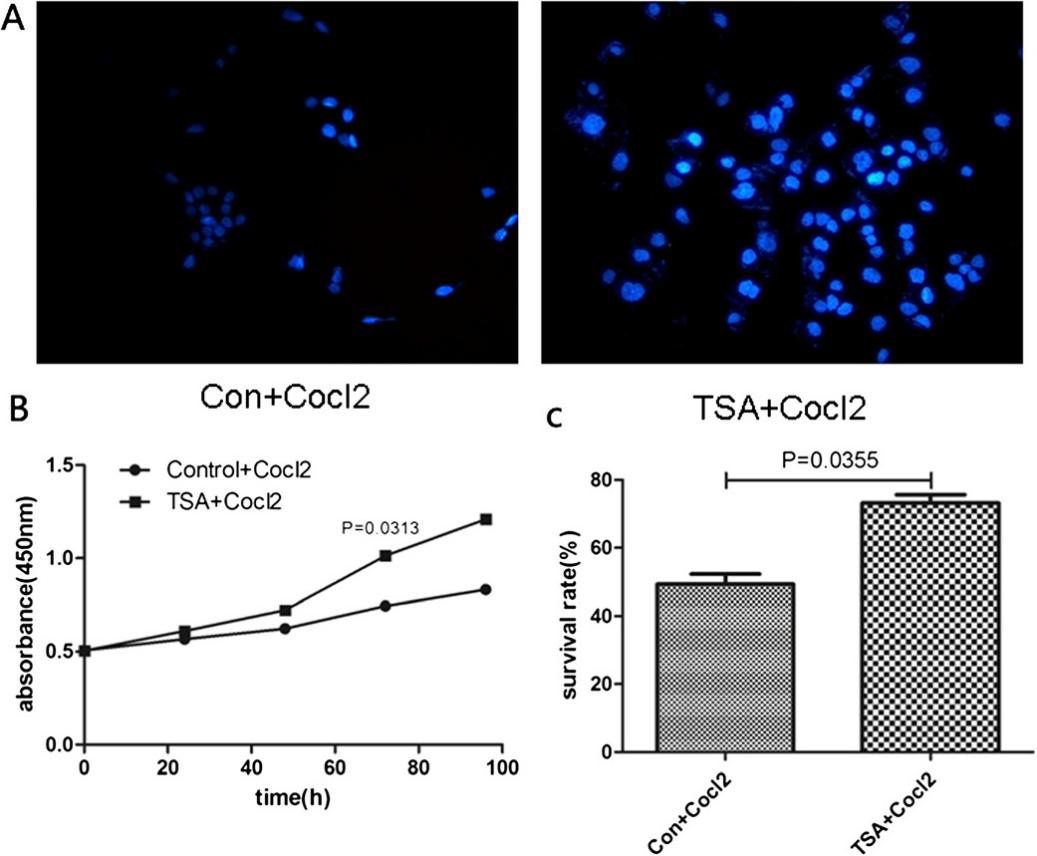
**Isolated epidermal cells that were pretreated with TSA for 2 weeks showed enhanced resistance to CoCl**_**2**_ **, which was used to mimic hypoxia** ***in vitro.*** After exposure to CoCl_2_ for 72 h, nuclear DAPI staining revealed larger number of TSA-pretreated cells than control cells **(A)**. A CCK-8 assay demonstrated that TSA-pretreated cells showed more proliferation than control cells after exposure to CgCl_2_ for 96 h **(B)**. A Trypan Blue assay showed that the survival rate of TSA-pretreated cells was higher than that of control cells after treatment with CoCl_2_ **(C)**.

DAPI staining showed that isolated epithelial skin cells that were pretreated with TSA (2 mg/L) for 2 weeks exhibited enhanced resistance to CoCl_2_. That is, the viability of control epithelial cells declined after 72 h of treatment with CoCl_2_, whereas epithelial cells pretreated with TSA showed higher viability (Figure 2A). A CCK-8 assay showed enhanced proliferation of TSA-pretreated cells after CoCl_2_ treatment compared with control cells (0.74 ± 0.02 vs. 1.01 ± 0.31, P = 0.0313; Figure 2B). Using a Trypan Blue Assay, we determined the exact proportion of viable epithelial cells after treatment with CoCl_2_ and found that the survival rate of TSA-pretreated epithelial cells was higher than that of control cells after treatment with CoCl_2_ (49.33 ± 7.29% vs. 73.17 ± 6.05%, P = 0.0355; Figure 2C).

### Epithelial skin cells showed upregulated Wnt signaling and increased expression of stem cell-related biomarkers after TSA pretreatment

**Figure 3:**
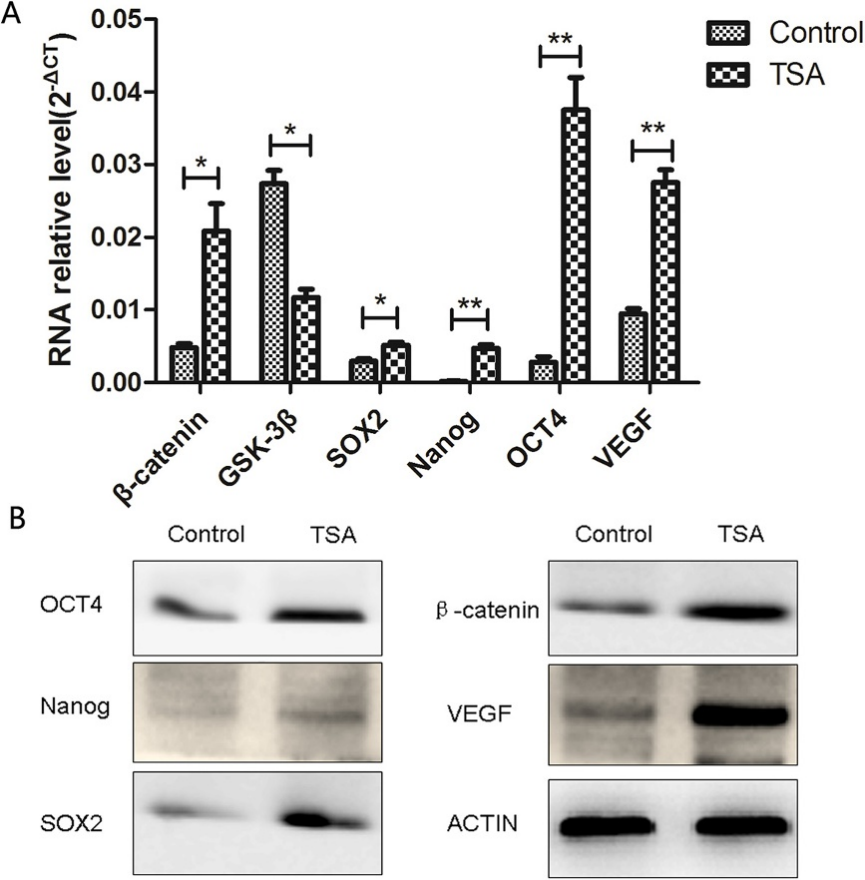
**Epidermal cells showed upregulated Wnt signaling and enhanced stemness after pretreatment with TSA.** qRT-PCR showed upregulation of β-catenin, SOX2, Nanog, OCT4, and VEGF and downregulation of GSK-3β in TSA-pretreated epithelial cells compared with control cells **(A)**. Similar results were obtained using western blot analysis **(B)**.

The Wnt signaling pathway is characterized by the expression of several biomarkers, such as GSK-3β and β-catenin, which are regarded as being critical for stem cell regulation. qRT-PCR revealed an upregulation of β-catenin (0.0048 ± 0.0005 vs. 0.0200 ± 0.0037, P = 0.014) and a downregulation of GSK-3β (0.0273 ± 0.0018 vs. 0.0117 ± 0.0012, P = 0.012) in TSA-pretreated epithelial cells compared with control cells (Figure 3A). The expression of stem cell markers SOX2 (0.0029 ± 0.0003 vs. 0.0051 ± 0.0004, P = 0.032), Nanog (0.0002 ± 0.0001 vs. 0.0046 ± 0.0005, P = 0.001), and OCT4 (0.0027 ± 0.0008 vs. 0.0375 ± 0.0045, P = 0.002) was higher in TSA-pretreated cells than in control cells. Furthermore, the expression of VEGF (0.0094 ± 0.0007 vs. 0.0276 ± 0.0018, P = 0.006) was higher in TSA-pretreated cells than in control cells. Similar results were found using western blot analysis (Figure 3B).

### TSA pretreatment reduced flap necrosis in an ischemic flap model

**Figure 4:**
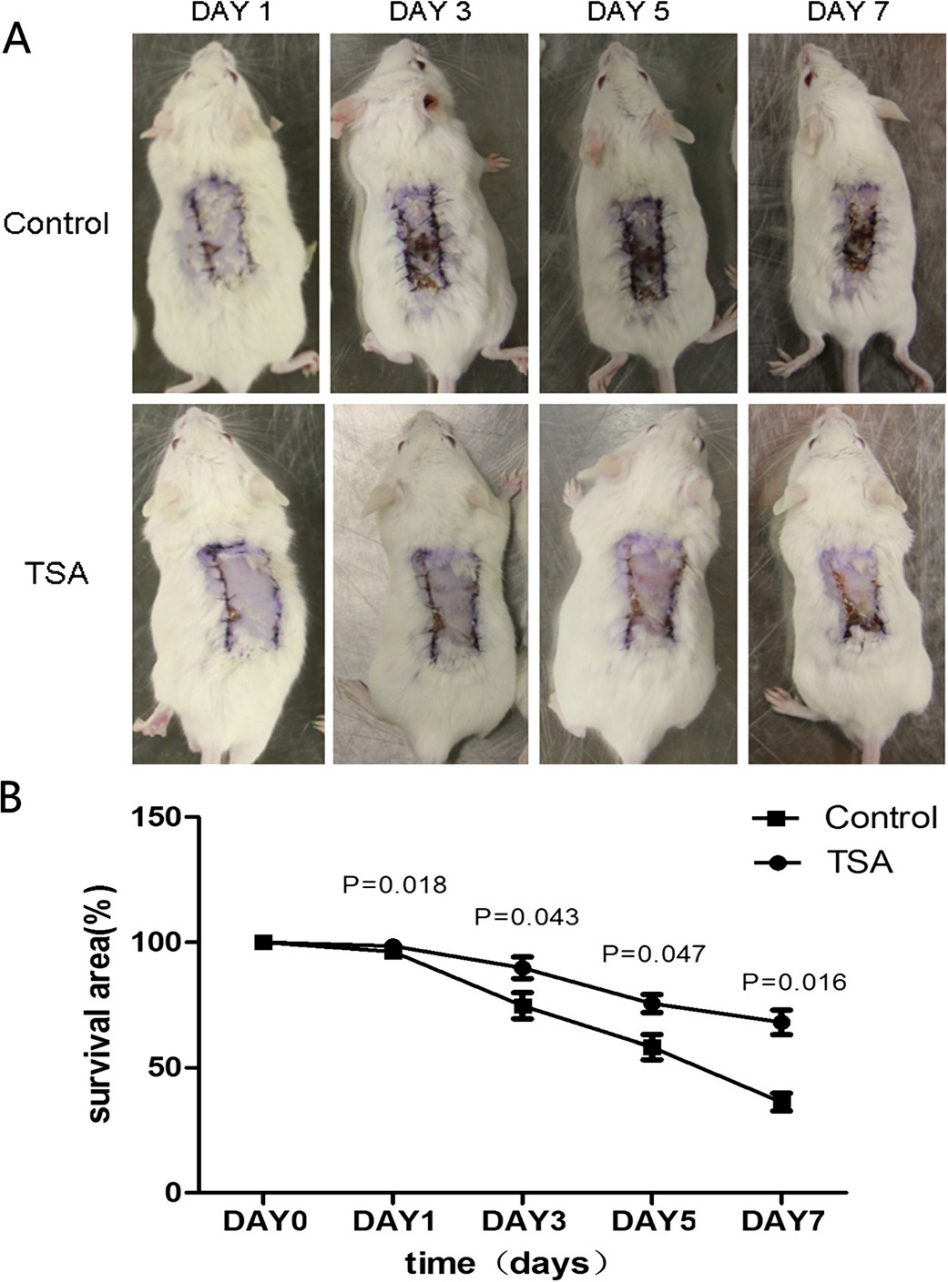
**Pretreatment with TSA enhanced resistance to hypoxia (A) and resulted in larger areas of surviving tissue (B) in an ischemic flap model.**

Pretreatment with TSA (10 mg/kg/d) enhanced tissue resistance to hypoxia and increased tissue survival area in an ischemic flap model (Figure 4A). That is, on the first day after surgery, there was no significant difference between TSA pretreatment and control groups in the area of surviving tissue (96.34 ± 2.19% vs. 98.56 ± 1.69%, P = 0.183; Figure 4B). On the third day, however, the ischemic flaps in the control group began to undergo necrosis, whereas the flaps in the TSA pretreatment group showed an enhanced resistance to hypoxia as evidenced by a greater tissue survival area (73.72 ± 6.09% vs. 89.82 ± 7.48%, P = 0.043). On the fifth day after surgery, flap necrosis in TSA-pretreated mice advanced more slowly than control mice (58.14 ± 8.67% vs. 75.54 ± 6.20%, P = 0.047). Finally, on the seventh day, flaps in TSA-pretreated mice showed a greater survival area than control mice (35.91 ± 10.22% vs. 68.02 ± 8.89%, P = 0.016).

### TSA treatment upregulated Wnt signaling and increased the expression of stem cell-related markers in epithelial skin tissue

**Figure 5:**
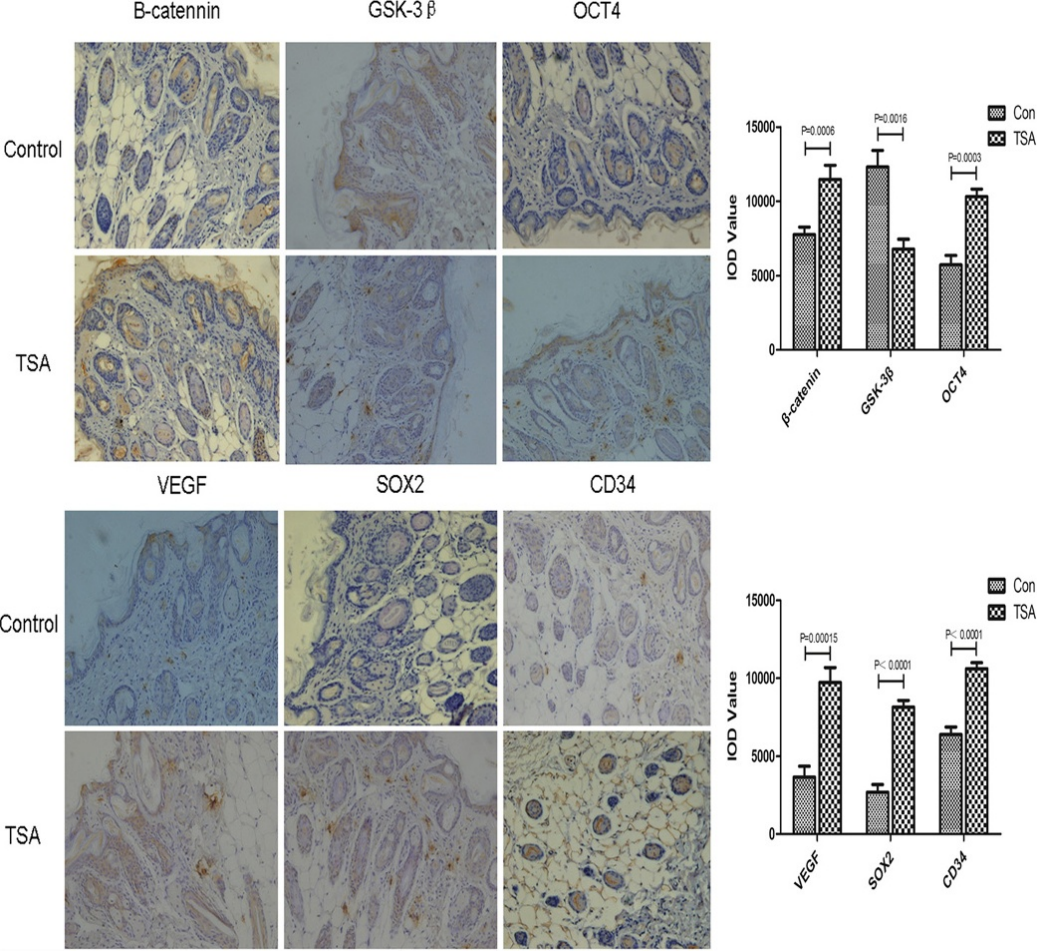
**TSA pretreatment upregulated Wnt signaling and increased the expression of stem cell-related markers in mouse epithelial skin tissue.**

To investigate the mechanism by which TSA protects against ischemia-induced flap necrosis, we examined components of the Wnt signaling pathway and stem cell-related biomarkers in epithelial skin tissue using immunohistochemistry. We observed increased expression of β-catenin and decreased expression of GSK-3β after TSA pretreatment (Figure 5). Stem cell-related biomarkers such as SOX2 and OCT4 were also upregulated after TSA pretreatment. Furthermore, the expression of VEGF, which is involved in the regeneration of blood vessels, and CD34, which is a marker of microvessel density, were also upregulated in epithelial skin tissue after pretreatment with TSA.

## Discussion

The human skin forms a large and important physical barrier between the body and its environment. In plastic surgery, tissue defects resulting from trauma, ablative surgery, or congenital malformation are frequently encountered, therefore flap transplantation is routinely used for re-establishing the epithelial barrier after injury. However, delayed healing or necrosis sometimes occurs when a free flap is transplanted from one area to another, which can increase the risk of infection and scar tissue formation or even lead to patient morbidity. Therefore, it is important for surgeons, who perform flap surgery, to have more knowledge of flap necrosis and how it can be prevented.

Ischemia is a condition of inadequate blood flow to a specific tissue area, which can lead to tissue hypoxia [11]. When the period of hypoxia exceeds the tolerance of transplanted free flap tissue, necrosis will occur. Whereas common epithelial skin cells show poor survivability of hypoxia, epithelial stem cells show a powerful resistance to maladaptive microenvironments [37]. In most organ systems, stem cells are thought to be the source of undifferentiated cells needed to maintain tissue homeostasis and to repair injury. One of the critical signaling pathways that regulates stem cell properties and plays an important role in skin organogenesis and regeneration is Wnt signaling, with several studies showing that Wnt signaling plays a critical role in tissue injury repair [21–23]. Phase I/II clinical trials demonstrate that enhancing Wnt signaling via antibody-mediated repression of Dkk1 is an effective means of stimulating new bone formation [38]. Also, in states of debilitating chronic injury, transiently elevating Wnt signaling is beneficial for tissue regeneration [39]. In the present experiment, we found that isolated epidermal cells that were pretreated with TSA showed upregulated Wnt signaling and enhanced resistance to hypoxia *in vitro*. We also found less necrosis and upregulated Wnt signaling in flap tissue after TSA pretreatment *in vivo*. Thus, although endogenous Wnt signaling is a prerequisite for tissue repair, there are obvious caveats to this general conclusion because of limited Wnt signaling activation in epithelial histiocytes [23, 40, 41]. Therefore, finding an effective and convenient way to pre-generate a sufficient amount of Wnt signaling activation may be important for injury restoration in hypoxic flaps.

Traditional Chinese medicine, a type of alternative and complementary medicine, is commonly used in Asian countries to treat cancer as well as cardiovascular, cerebrovascular, metabolic, and neurodegenerative diseases. TSA, which is isolated from Danshen (*Salvia miltiorrhiza*), has multiple targets including transcription factors, scavenger receptors, ion channels, kinases, pro- and anti-apoptotic proteins, growth factors, inflammatory mediators, and microRNA [42]. For instance, Tang et al. showed that the neuroprotective role of TSA monotherapy is mediated by the PI3K/AKT signaling pathway [43]. Chen et al. found that TSA alleviates the proinflammatory responses associated with I/R-induced injury by downregulating MIF expression [24]. In addition, Zhang et al. showed that TSA enhances cell resistance to hypoxic insult by upregulating miR-133 expression through activation of MAPK-ERK1/2 [31]. In the present study, we found that epithelial skin cells, pretreated with, TSA showed greater viability than control cells after CoCl_2_ treatment *in vitro*. We also found that pretreatment with TSA enhanced tissue resistance to hypoxia and resulted in larger areas of surviving tissue in an *in vivo* ischemic flap model. Furthermore, we demonstrated that activation of the Wnt signaling pathway with the increased expression of β-catenin and down-regulation of GSK-3β. The increased expression of stem cell-related markers such as SOX2, OCT4, and Nanog were also involved in the effect of TSA on epithelial cells and tissue with the activation of the Wnt signaling pathway. As to VEGF which is the base of angiogenesis was also up-regulated involve in the effect of TSA. So, we also found the increased expression CD34 after treated by TSA. And all of these stem cell- and angiogenesis -related markers determined the survival of flap during the process of ischemia and hypoxia after injury.

In summary, we provide experimental evidence that TSA pretreatment protects against necrosis of transplanted flap tissue in mice, suggesting that TSA pretreatment might be a potential way to improve the success of free flap surgery in humans. However, several fundamental questions remain concerning the ability of TSA to increase the stemness of epithelial skin cells. Therefore, additional animal models of ischemia should be utilized to further investigate the relationship between TSA and stemness, and future studies could also determine whether TSA can be clinically useful for treating human ischemic tissues and organs.

## Conclusions

TSA pretreatment protects free flaps against hypoxic injury and increases the area of surviving tissue in transplanted flaps by activating Wnt signaling and upregulating stem cell-related biomarkers in epithelial skin cells.

## Abbreviations

TSA: Tanshinone II-A
I/R: Ischemia/reperfusion
MIF: Macrophage migration inhibitory factor
DAPI: 4’-6-Diamidino-2-phenylindole
CoCl_2_: Cobalt chloride
VEGF: Vascular endothelial growth factor
SOX2: Sex determining region Y-box 2
CCK-8: Cell Counting Kit-8
GSK-3β: Glycogen synthase kinase-3 beta
MAPK: Mitogen-activated protein kinase
ERK: Extracellular signal-related kinase
PBS: Phosphate-buffered saline
DMEM: Dulbecco’s Modified Eagle’s Medium
DAPI: 4’-6-Diamidino-2-phenylindole
qRT-PCR: Quantitative real-time polymerase chain reaction.
